# Concentric-Electrode Organic Electrochemical Transistors: Case Study for Selective Hydrazine Sensing

**DOI:** 10.3390/s17030570

**Published:** 2017-03-11

**Authors:** Sébastien Pecqueur, Stéphane Lenfant, David Guérin, Fabien Alibart, Dominique Vuillaume

**Affiliations:** Institut d’Electronique, Micro-électronique et Nanotechnologie, CNRS, CS 60069, Avenue Poincaré, 59652 Villeneuve d’Ascq CEDEX, France; stephane.lenfant@iemn.univ-lille1.fr (S.L.); david.guerin@iemn.univ-lille1.fr (D.G.); fabien.alibart@iemn.univ-lille1.fr (F.A.); dominique.vuillaume@iemn.univ-lille1.fr (D.V.)

**Keywords:** OECT, PEDOT:PSS, hydrazine sensor, organic electronics, microelectronics

## Abstract

We report on hydrazine-sensing organic electrochemical transistors (OECTs) with a design consisting of concentric annular electrodes. The design engineering of these OECTs was motivated by the great potential of using OECT sensing arrays in fields such as bioelectronics. In this work, poly(3,4-ethylenedioxythiophene):poly(styrenesulfonate) (PEDOT:PSS)-based OECTs have been studied as aqueous sensors that are specifically sensitive to the lethal hydrazine molecule. These amperometric sensors have many relevant features for the development of hydrazine sensors, such as a sensitivity down to 10^−5^ M of hydrazine in water, an order of magnitude higher selectivity for hydrazine than for nine other water-soluble common analytes, the capability to entirely recover its base signal after water flushing, and a very low operation voltage. The specificity for hydrazine to be sensed by our OECTs is caused by its catalytic oxidation at the gate electrode, and enables an increase in the output current modulation of the devices. This has permitted the device-geometry study of the whole series of 80 micrometric OECT devices with sub-20-nm PEDOT:PSS layers, channel lengths down to 1 µm, and a specific device geometry of coplanar and concentric electrodes. The numerous geometries unravel new aspects of the OECT mechanisms governing the electrochemical sensing behaviours of the device—more particularly the effect of the contacts which are inherent at the micro-scale. By lowering the device cross-talk, micrometric gate-integrated radial OECTs shall contribute to the diminishing of the readout invasiveness and therefore further promote the development of OECT biosensors.

## 1. Introduction

Polymeric-semiconductor devices have already shown great promise to substitute many technologies behind consumer products such as electronics [1], photovoltaics [2], lighting [3], and displays [4]. Recently, efforts have been devoted to employ the outstanding features of these plastic electronics technologies to create smarter sensors, such as electrolyte-gated organic field-effect transistors [5] or organic photodetectors [6]. More specifically, the rich chemistry of molecular and polymeric semiconductors offers the possibility of building biocompatible sensors. Indeed, organic electrochemical transistors are a type of electrolyte-gated transistors in which the constituting semiconducting ionomer blend has a good affinity with cations. As such, this platform is extensively studied as a bio-electronical interface to measure neural activity [7,8,9]. Nowadays, one of the key challenges for this technological tool is to interpret complex readouts of dense networks. To control a whole array, one should first engineer the organic electrochemical transistor (OECT) units, and second understand the mechanism of these units that is responsible for their sensitivity.

Efforts have been devoted to the theoretical study of capacitive sensors having a radial symmetry [10], but these geometries are not extensively applied for practical applications. Here, we report results on OECTs with a circular geometry offering a higher sensing isotropy and a large geometry variation to interpret the device functioning. Additionally, the whole device comprises its individual gate electrode, which is locally patterned on the substrate in an overall diameter down to 100 µm. Despite the high current densities obtained at very low operating voltage, the dimension restrictions strongly limit the active areas of the sensor, which hinders its gate-controlled response. Therefore, we activated the large-gate output-current modulation by the introduction of hydrazine. The OECTs have been shown to be highly sensitive and selective to hydrazine. Hydrazine is a 0.1-ppm-level genotoxic, and while the human carcinogenicity has not been evidenced, high caution is still recommended on inhalation-induced lung-cancer risks [11]. Thus, the ability to realise this kind of sensor is then of the clearest interest. Moreover, these OECTs have shown various performances depending on their geometry, and in this study we have explored the hydrazine-enabled gate OECT on a population of 80 devices with different geometries to better understand their functioning.

## 2. Materials and Methods

**Figure 1:**
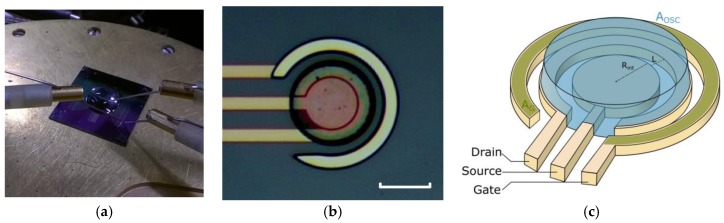

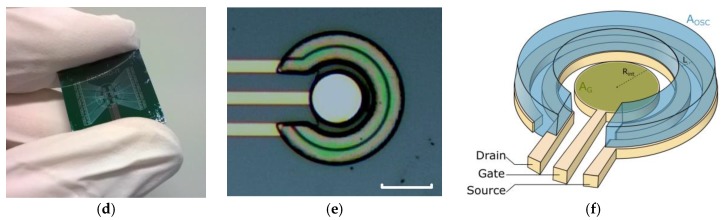
(**a**,**b**) Electrical setup including the 4 cm^2^ organic electrochemical transistor (OECT)-patterned silicon substrate with the electrolyte drop dispensed on top of it. (**c**,**e**) Source-centred and (**d**,**f**) gate-centred OECTs: (**c**,**d**) Device microscope pictures (scale bars: 40 µm) and (**e**,**f**) depiction of their architecture—the representation of the passivating parylene C has been omitted for the sake of clarity).

The devices have been fabricated by e-beam lithography (see Figure 1a–d), adapted from a previously-reported process [12]. Platinum electrodes have been patterned by e-beam lithography and lift-off on top of a 200 nm thermally-grown SiO_2_/n-type Si substrate, using a resist of poly(methyl methacrylate). Ten nanometres of titanium were evaporated as an adhesion layer prior to the 70 nm of platinum. Platinum was chosen as a contact metal for both source/drain and gate because of its chemical inertia and its high work-function forming ohmic contacts with organic hole transport materials. Then, SiO_2_ was functionalized with (3-glycidyloxypropyl)trimethoxysilane (22 mM-concentrated in a 40 mL 200:1 toluene:acetic-acid solution) as an adhesion promoter, prior to the processing of a 2-µm-thick poly(chloro-para-xylylene) (or parylene C) capping layer by chemical vapour deposition using the Gorham process (dichloro[2,2]-para-cyclophane was used as a precursor in a C20S deposition chamber from Comelec). Then, the device apertures were patterned by e-beam lithography with a sacrificial 3.7-µm-thick resist (a modified UV210 from Rohm and Haas Electronic Materials LLC) before dry-etching the surface with an oxygen plasma. Then, a surfactant (2% *v/v* Micro-90 in deionized water) was dispensed on top of the substrate by spin-coating, before depositing a subsequent 2-µm-thick parylene C layer (under similar conditions as previously). Another e-beam lithography and dry-etching step with similar conditions as previously laid out was carried out in order to etch the channel cavities and mask the gate apertures. The channel cavities were filled by a filtered and spin-coated PEDOT:PSS solution (76% *v/v* Clevios PH1000 Heraeus, 19% *v/v* ethylene glycol, 4% *v/v* dodecylbenzenesulfonic acid, 1% *v/v* (3-glycidyloxypropyl)trimethoxysilane) and the underlying parylene C layer was carefully stripped out with some adhesive tape before an hour long hard baking at 140 °C.

All the electrical characterizations were performed on an Agilent 4155 parameter analyser at a scan rate of 5 mV/s, OECTs grounded at the gate. Before measurements and to ensure the highest reproducibility [13], the PEDOT:PSS patches were stressed three times with a grounded platinum wire gate at −700 mV in a 1 M KCl aqueous solution (sweeps from 0 to −700 mV on a 5 mV/s scan rate). Although the potentials are not referenced, we set the highest polarisation to 700 mV in absolute value, Pt gate grounded, in order to lower the risk of water electrolysis (no significant steady-state current density at the gate electrode was observed in the study).

## 3. Selectivity/Sensitivity Study for a Single Hydrazine Sensor OECT

Central symmetry OECTs have been fabricated on either a source-centred (the term “source-centred” refers strictly to a device geometry and not the polarisation of the source with respect to the drain—this information is specified by the term “direct polarisation”, as opposed to “reversed polarisation”) or gate-centred configuration (see Figure 1e,f). The geometry of the devices was modulated by the channel length L (20, 10, 5, 2, and 1 µm) and the radius of the central electrode R_int_ (50, 40, 30, and 20 µm). Practically, the round-shaped PEDOT:PSS patches on the source-centred OECTs were patterned more easily than the “C”-shaped ones on the gate-centred OECTs, which required a preferential direction for the parylene C peeling as well as a fine tuning of the baking and peeling conditions to have an optimised yield.

**Figure 2:**
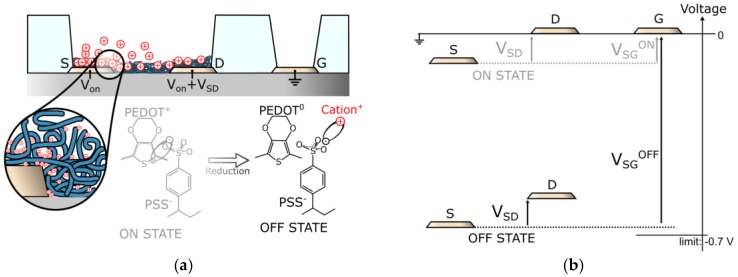

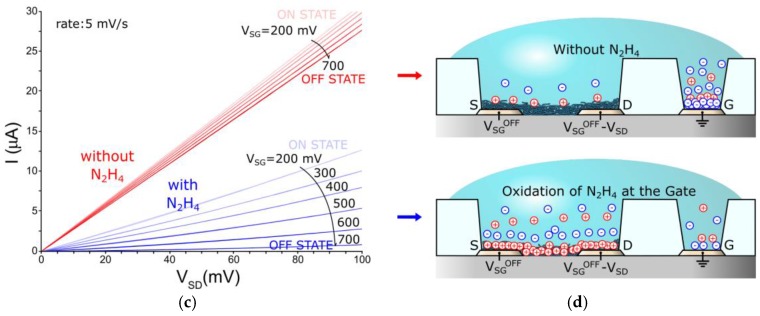
Behaviour of the OECT: (**a**) Sketch of the widely-accepted OECT operation model, implicating the participation of cations in the PEDOT dedoping mechanism upon channel/gate polarization; (**b**) Diagram of the applied voltage biases, necessary in our experiments to read the OECT ON and OFF states; (**c**) Typical current–voltage characteristics of an OECT without and with hydrazine (conditions: 1 M KCl, 0.1 M N_2_H_4_, source-centred, L = 5 µm, and R_int_ = 20 µm); (**d**) Schematic of two cases of different ionic distributions on an OECT under operation. On the top, the case for which the voltage drops mainly at the gate/electrolyte interface. On the bottom, the case for which the voltage drops mainly at the PEDOT/electrolyte interface.

The PEDOT:PSS patches overlap the whole top area of the source and drain electrodes on a width of at least 10 µm in order to ensure the maximal injection of current in the channel. Based on a well-accepted mechanism [14], the channel conductance can be modulated upon source-and-drain/gate polarisation, such as the PEDOT semiconductor is dedoped from the PSS by reduction, supported by cations which penetrate the ionomer and chelate the sulfonates (see Figure 2a,b). Both the presence of labile cations and the device polarisation are essential but not sufficient in order to dedope the PEDOT.

Indeed, the first tests on our OECTs in hydrazine-free aqueous electrolytes of KCl showed weak conductance modulations in the studied voltage windows, even for concentrations up to 10 M of KCl (see Figure 2c). As published, the OECT dedoping efficiency depends on multiple parameters other than ionic concentration and applied voltages, such as the ratio of the channel area (A_ch_) over the area of the gate (A_g_) [15,16], the nature of the gate metal [17], and also the volume of PEDOT:PSS to dedope [18,19]. In order to achieve a significant dedoping of the whole PEDOT:PSS material with a small gate, the volume of PEDOT:PSS was kept as low as possible. Layers thinner than 20 nm of PEDOT:PSS were realised by spinning the formulation at velocities higher than 4000 rpm, followed by a subsequent spinning of deionised (DI) water to thin it further before substrate baking. Despite the small thickness of polymer, rather good performances were obtained, reaching currents up to 60 µA. Additionally, the lack of hysteresis and the linearity of the curves indicate, respectively, a rapid dedoping at a scan rate of 5 mV/s and that the currents are ohmic (see Figure 2c).

Despite the low volume of PEDOT:PSS, the usage of Pt as gate metal and the small A_ch_/A_g_ poorly modulated the output-current density. It has been proposed that the electrochemical inertia of the gate metal in addition to its small size is the origin of the lack of field-effect, causing the voltage to drop mainly at the platinum gate interface, screening the channel from polarisation [17] (see Figure 2d, upper sketch). Additionally, in order to counterbalance this effect for the same A_ch_/A_g_, strategies such as using a fast redox-system gate like Ag/AgCl [17] or introducing a redox active analyte such as H_2_O_2_ in the electrolyte [15] have been used. In the case of H_2_O_2_ introduction, it has been reported that the field-effect on the OECT depends on the concentration of H_2_O_2_ introduced in the electrolyte—the OECT can be used as a H_2_O_2_ sensor down to 10^−5^ M (level of mg/L) [15].

In this study, we demonstrate the OECT to be far more selective to hydrazine. As a strong reductant, its electro-catalysed oxidation at the Pt gate supports the reduction of the PEDOT at the channel upon polarisation, creating N_2_ and N_2_H_5_^+^ as byproducts (the nature of these byproducts is highly pH-dependent) [20]. Considering the oxidation products which do not deposit on the gate, the chemical potential at the Pt-gate shall remain stable over time, and the mirrored potential shall not depend on the sensor usage.

**Figure 3:**
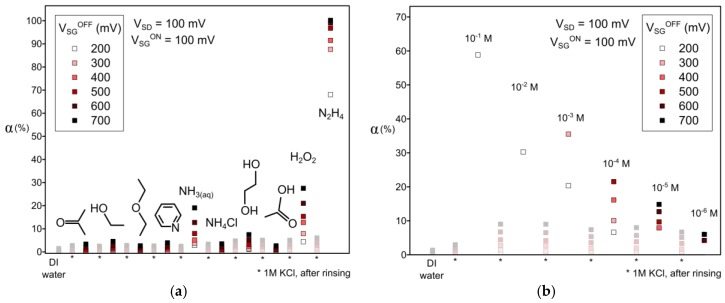
Selectivity and sensitivity of an OECT (conditions: source-centred, L = 5 µm, and R_int_ = 20 µm) to hydrazine at different source-gate potentials: (**a**) Study of different 0.1 M concentrated analytes diluted in 0.1 M KCl solutions; (**b**) Study of N_2_H_4_ diluted in 1 M KCl solutions. Data on each graph are displayed from left to right in chronological order.

The electrical measurements of a source-centred OECT were performed with N_2_H_4_ at different electrical and chemical conditions, after the device was immersed overnight in deionised water (see Figure 3).

If α(V_SG_^OFF^, V_SG_^ON^, V_SD_) is the amplitude of current modulation between the off-state and the on-state, defined as:

(1) $$\alpha =\frac{I\left(V_{SG}{}^{ON};V_{SD}\right)-I\left(V_{SG}{}^{OFF};V_{SD}\right)}{I\left(V_{SG}{}^{ON};V_{SD}\right)}=1-\frac{I_{OFF}}{I_{ON}}$$

and fixing the values V_SD_ and V_SG_^ON^ at 100 mV, the sensor shows current density modulations α(V_SG_^OFF^) below 3% with deionised water and 1 M concentrated KCl (see Figure 3). This demonstrates that the output is barely sensitive to the concentration of KCl in the absence of an electroactive analyte. Different analytes were successively introduced and tested on the OECT, followed by a rinse and test on a 1 M KCl concentrated blank (see Figure 3a). After each analyte test, the sensor recovered down to a α = 5 ± 2 at V_SG_^OFF^ = 700 mV, indicating that the OECT was not damaged by any analyte. The sensor displayed α for acetone, ethanol, diethylether, pyridine, ammonium chloride, and acetic acid very near that of the blanks, showing the insensitivity of the OECT for 0.1 M of these analytes in water. A slight modulation with 0.1 M of ethylene glycol was observed, but without significant change in the PEDOT:PSS current density (since this analyte has the property to boost the conductivity of PEDOT:PSS [21], the lack of sensitivity to this molecule is of the highest importance). Moderated modulations with 0.1 M of aqua ammonia or hydrogen peroxide were observed. Confirming the applicability of H_2_O_2_ sensing with an OECT, it is worth noticing that other metabolites such as ammonia can also be detected at the same level. While 0.1 M of ammonia or hydrogen peroxide modulate with α = 25% ± 5% at V_SG_^OFF^ = 700 mV, hydrazine promotes α = 65% ± 5% at as few as V_SG_^OFF^ = 200 mV at the same concentration. The channel is completely closed with I_OFF_ = 3 ± 2 nA when V_SG_^OFF^ = 700 mV, resulting to α ≈ 100%.

Dry hydrazine has the property to reduce p-doped polythiophenes such as P3HT [22]. An eventual thermodynamically-favoured reduction of the PEDOT by N_2_H_4_ could explain the observed decrease of I_off_ with the concentration of N_2_H_4_ (see Figure 2a). Nevertheless, this single property does not sufficiently justify the V_SG_^OFF^ dependency of the PEDOT conductivity, since the electrode polarisation under device operation promotes the electrolyte oxidation at the gate electrode and not at the PEDOT channel. The OECT sensor also presents an acute sensitivity to hydrazine (see Figure 3b). Successive dilutions of fresh hydrazine aqueous KCl solutions show the sensor to be sensitive to at least 10^−5^ M of N_2_H_4_ at the studied voltages. The sensor responds positively for an applied V_SG_ from 200 mV to 700 mV when exposed to an N_2_H_4_ electrolyte of concentrations from 0.1 M to 10^−5^ M. The response α remains at the base level when immersed in a 10^−6^ M concentrated N_2_H_4_ electrolyte. We note that the sensor regeneration to α = 9 ± 1 strongly limits the device sensitivity at 10^−6^ M. Another operation protocol on the device rinsing step or on the electrical inputs could be thought to improve the sensor regeneration and therefore to increase the sensitivity range of the sensor, but it has not been explored in this work.

## 4. Structure–Property Study of a Population of 80 OECTs

Studies on millimetre-scale OECTs demonstrated that the sensitivity α of the devices to ions was highly dependent on gate electrode area [15]. Therefore, a parameter γ = A_ch_/A_g_ was defined as a preferred geometry parameter for the sensitivity of OECTs to ions at a given concentration and voltage [15]. Although this study enabled the correlation of the device geometry to a dedoping strictly occurring in the channel, most practical micrometric bottom-contacts transistors require a large area for the source-and-drain contacts to be exposed to the electrolyte [7]. This implies that the contact area must be considered as counter-electrode for the gate, and therefore impacts α by both dedoping of the channel and the contacts, affecting both the channel conductivity and the Schottky barriers at the contacts. Since the conductivity and the Schottky barrier have a different dependency on the hole carrier density in the PEDOT [23], we believe that α of the OECTs can be severely impacted by parameters which are not exclusively channel-related, such as the contacts geometry.

**Figure 4:**
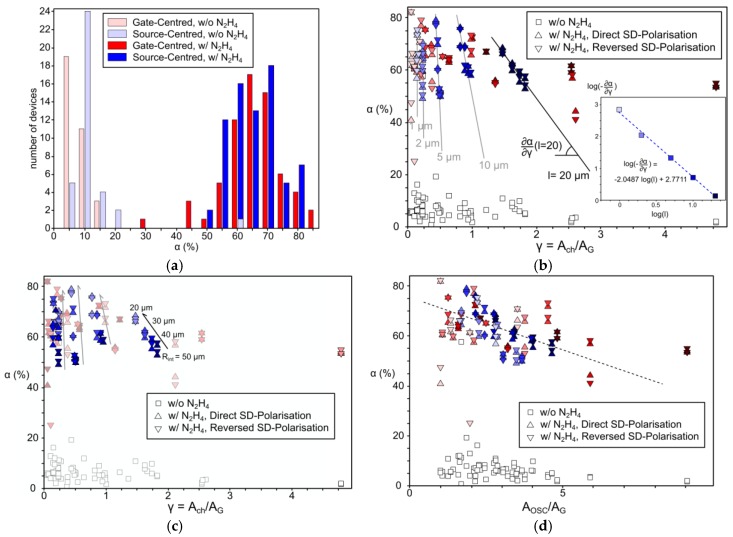
Study of the geometry-dependent sensitivity of OECTs: (**a**) Statistical study on the sensitivity α for all geometries of OECTs, with and without N_2_H_4_; (**b**) Plot of α(γ) showing the channel length dependency of the derivative for the source-centred OECTs (the inset plot shows the quadratic dependency); (**c**) Plot of α(γ) showing the dependency of α with the radius of the central electrode for the source-centred OECTs; (**d**) Plot of α(A_OSC_/A_G_) showing a better correlation when considering a contact-area-dependent parameter (the dashed line is a guide for the eye). For all graphs, red accounts for gate-centred OECTs and blue for source-centred ones. In (b,d), the colour gradients are such as the lighter, the shorter channel and for (c) the lighter, the smaller the central electrode.

To characterise it, tests of 10^−2^ M N_2_H_4_ in 10^−1^ M KCl_aq_ were realised for all device geometries, with V_SG_^ON^ = 700 mV and V_SG_^OFF^ = V_SD_ = 100 mV (see Figure 4). All devices showed a sensitivity α from 30% to 85% for both source-centred geometries and gate-centred ones (see Figure 4a). When referenced to 1 M KCl_aq_, one sees the base modulation α without hydrazine to be located between 0% and 20% for both the source-centred and the gate-centred OECTs.

When plotting the sensitivity α against the geometric γ parameter of each device (see Figure 4b), one can distinguish a different amplitude for α of each OECT between operation with hydrazine and without hydrazine, showing all the devices to be operational and sensitive to it. Additionally, α of the OECTs with reversed SD-polarisation are similar to those of the direct SD-polarisation, indicating the insensitivity of α to the surface area of the hole injecting electrode, even for the largest R_int_ source-centred devices. For the source-centred, a trend for α to decrease linearly with γ can be seen for a given channel length, and the slope of this trend seems to be channel-length dependent.

When plotting the logarithm of the slope for each |α|(γ) curves and for the different source-centred channel lengths, a slope equal to −2 can be seen (see the inset graph of Figure 4b), indicating that the source-centred OECTs have a sensitivity particularly dependent on the channel length, such that the variation of α with γ increases in absolute value with 1/L^2^. However, the fact that the five series of different channel lengths with different γ have comparable values of α under the same electrochemical conditions indicates that α depends on a geometric constraint other than γ = A_ch_/A_G_. When looking at the radius of the central electrode for each channel-length series of source-centred OECTs (see Figure 4c), one sees a monotonic trend for the sensitivity α to increase when the radius of the central electrode decreases. This indicates α to be source-and-drain-contact-area dependent, and therefore the overlapping of the PEDOT:PSS with the contacts shall be considered for the design of electrochemical OECT sensors.

As an example, Figure 4d shows that when we include the contact area in the discriminating geometric parameter, we see that the series for both gate-centred and source-centred OECTs have the same sensitivity trends, with an overall trend for α to decrease with the area ratio PEDOT:PSS/gate (A_OSC_/A_G_).

In the end, α for the gate-centred series remain more dispersed around the trend than the source-centred ones, when plotted against either γ or A_OSC_/A_G_. This dispersion of α values for the gate-centred devices do not allow us to interpolate obvious trends with the channel lengths or the contact area as for the source-centred devices. Since the dimensions of both geometries are very comparable to each other (and thus their γ and A_OSC_/A_G_ values), we believe the “C”-shaped PEDOT:PSS patches on the gate-centred devices are more subject to variabilities than the round ones on the source-centred.

Regarding the trend for α to increase with the decrease of the contact area for source-centred OECTs, we would also recommend to diminish it to further increase the OECT sensitivity.

## 5. Conclusions

We report both a technological and a scientific result in the field of PEDOT:PSS-based organic electrochemical transistors for sensing. First, we demonstrate that this platform can selectively sense hydrazine, and as such, can be a promising structure to sense toxics in liquids. Second, a structure–property relationship showed the OECT to be highly contact sensitive. Future investigations on models considering the contact area of the devices in their functioning could help us to further understand their mechanism. This whole study was performed on micrometric concentric electrode OECTs—a set of engineered devices which might promote the development of higher definition electrochemical transistors.

## Supplementary Materials

The following are available online at http://www.mdpi.com/1424-8220/17/3/570/s1.
