# Supplementary material for: Concentric-Electrode Organic Electrochemical Transistors: Case Study for Selective Hydrazine Sensing
Source: Sensors (Basel). 2017 Mar 11;17(3):570. doi: 10.3390/s17030570 (PMC5375856; doi:10.3390/s17030570)
Supplement: Supplementary file 1 [file sensors-17-00570-s001.pdf]

Supplementary Informations

Concentric-electrode organic electrochemical transistors: case study for selective hydrazine sensing

Sébastien Pecqueur [1,*], Stéphane Lenfant [1], David Guérin [1], Fabien Alibart [1] and Dominique Vuillaume [1]

1 Institut d'Electronique, Micro-électronique et Nanotechnologie, CNRS, CS 60069, avenue Poincaré, 59652 Cedex, Villeneuve d'Ascq, France

* Correspondence: sebastien.pecqueur@iemn.univ-lille1.fr; Tel.: +33-320-19-79-79

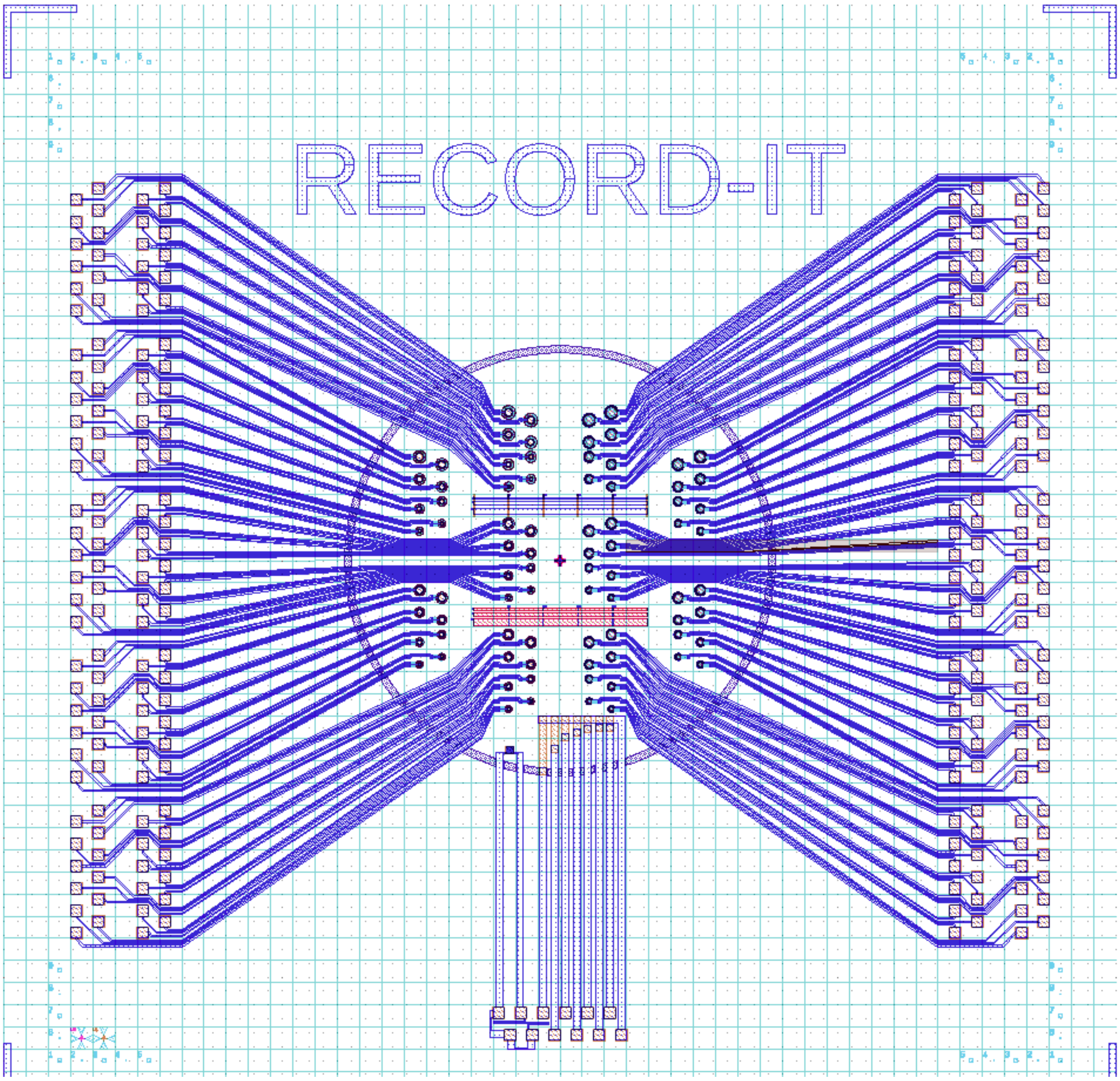

Figure S1. Susbtrate layout for the OECTs studied

Device #	Geometry	Channel (µm) Length	Width*	W/L	2·Rint (µm)	S(S) (µm²)	S(D) (µm²)	S(G) (µm²)	S(OSC) (µm²)	S(Ch) (µm²)	γ'= S(OSC)/S(G)	γ= S(Ch)/S(G)
1, 2	Gate-cent.	20	489	24	100	4672	3589	7533	17461	9200	2.32	1.22
3, 4	Gate-cent.	20	427	21	80	4100	3026	4772	15070	7944	3.16	1.66
5, 6	Gate-cent.	20	364	18	60	3532	2473	2639	12714	6709	4.82	2.54
7, 8	Gate-cent.	20	302	15	40	2935	1901	1133	10265	5430	9.06	4.79
9, 10	Gate-cent.	10	464	46	100	4206	3495	7533	11986	4285	1.59	0.57
11, 12	Gate-cent.	10	401	40	80	3065	2931	4772	10223	4227	2.14	0.89
13, 14	Gate-cent.	10	338	34	60	3065	2370	2639	8455	3020	3.20	1.14
15, 16	Gate-cent.	10	275	27	40	2478	1801	1133	6677	2398	5.89	2.12
17, 18	Gate-cent.	5	451	90	100	3973	3448	7533	9484	2063	1.26	0.27
19, 20	Gate-cent.	5	389	78	80	3403	2883	4772	8035	1749	1.68	0.37
21, 22	Gate-cent.	5	325	65	60	2831	2318	2639	6582	1434	2.49	0.54
23, 24	Gate-cent.	5	262	52	40	2250	1752	1133	5119	1117	4.52	0.99
25, 26	Gate-cent.	2	444	222	100	3832	3420	7533	8058	806	1.07	0.11
27, 28	Gate-cent.	2	381	191	80	3263	2855	4772	6798	680	1.42	0.14
29, 30	Gate-cent.	2	318	159	60	2691	2289	2639	5534	554	2.10	0.21
31, 32	Gate-cent.	2	254	127	40	2112	1721	1133	4260	427	3.76	0.38
33, 34	Gate-cent.	1	442	442	100	3785	3410	7533	7596	400	1.01	0.05
35, 36	Gate-cent.	1	379	379	80	3216	2845	4772	6398	337	1.34	0.07
37, 38	Gate-cent.	1	315	315	60	2645	2279	2639	5197	274	1.97	0.10
39, 40	Gate-cent.	1	251	251	40	2066	1710	1133	3987	210	3.52	0.19
41, 42	Source-cent.	20	351	18	100	8134	3749	4216	19582	7699	4.64	1.83
43, 44	Source-cent.	20	289	14	80	5310	3181	3709	14938	6447	4.03	1.74
45, 46	Source-cent.	20	227	11	60	3114	2611	3199	10922	5197	3.41	1.62
47, 48	Source-cent.	20	166	8	40	1546	2035	2683	7533	3952	2.81	1.47
49, 50	Source-cent.	10	322	32	100	8034	3324	3755	14938	3581	3.98	0.95
51, 52	Source-cent.	10	260	26	80	5210	2757	3248	10922	2955	3.36	0.91
53, 54	Source-cent.	10	197	20	60	3014	2189	2737	7533	2330	2.75	0.85
55, 56	Source-cent.	10	135	13	40	1446	1617	2218	4772	1710	2.15	0.77
57, 58	Source-cent.	5	310	62	100	7984	3087	3502	12852	1781	3.67	0.51
59, 60	Source-cent.	5	247	49	80	5160	2520	2993	9149	1469	3.06	0.49
61, 62	Source-cent.	5	184	37	60	2964	1951	2479	6074	1159	2.45	0.47
63, 64	Source-cent.	5	120	24	40	1396	1376	1951	3627	856	1.86	0.44
65, 66	Source-cent.	2	302	151	100	7954	2944	3350	11675	777	3.49	0.23
67, 68	Source-cent.	2	239	120	80	5130	2377	2840	8161	654	2.87	0.23
69, 70	Source-cent.	2	176	88	60	2934	1807	2329	5274	533	2.26	0.23
71, 72	Source-cent.	2	110	55	40	1366	1227	1784	3015	423	1.69	0.24
73, 74	Source-cent.	1	300	300	100	7944	2896	3299	11295	455	3.42	0.14
75, 76	Source-cent.	1	237	237	80	5120	2329	2789	7844	394	2.81	0.14
77, 78	Source-cent.	1	173	173	60	2924	1759	2271	5020	337	2.21	0.15
79, 80	Source-cent.	1	106	106	40	1356	1176	1726	2824	292	1.64	0.17

*measured half way between the source and the drain

Figure S2. Geometrical properties of the 80 different OECTs

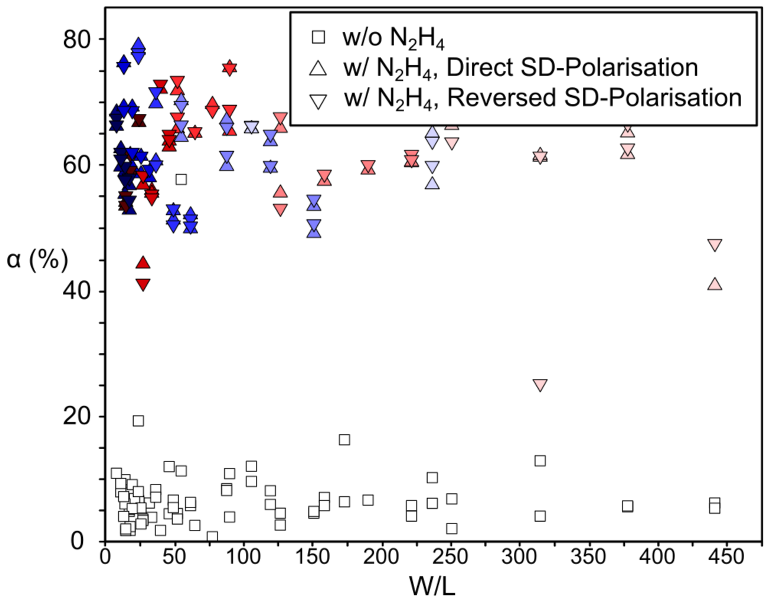

Figure S3. Plot of the current modulation parameter α vs. the channel width to length ratio W/L, showing no clear coupling between the effect of the channel on the device sensitivity for these large contacts OECTs. Red accounts for gate-centred OECTs and blue for source-centred ones, the lighter the colour, the shorter channel
